# 中国华氏巨球蛋白血症诊断与治疗现状调查：一项横断面研究

**DOI:** 10.3760/cma.j.cn121090-20231017-00212

**Published:** 2024-02

**Authors:** 树华 易, 文婕 熊, 欣欣 曹, 春艳 孙, 鹃 杜, 慧涵 王, 黎 王, 挺 牛, 中兴 姜, 永强 魏, 华 薛, 红玲 褚, 录贵 邱, 剑 李

**Affiliations:** 1 中国医学科学院血液病医院（中国医学科学院血液学研究所），血液与健康全国重点实验室，国家血液系统疾病临床医学研究中心，细胞生态海河实验室，天津 300020 State Key Laboratory of Experimental Hematology, National Clinical Research Center for Blood Diseases, Haihe Laboratory of Cell Ecosystem, Institute of Hematology & Blood Diseases Hospital, Chinese Academy of Medical Sciences & Peking Union Medical College, Tianjin 300020, China; 2 天津医学健康研究院，天津 301600 Tianjin Institutes of Health Science, Tianjin 301600, China; 3 中国医学科学院北京协和医院，北京 100730 Peking Union Medical College Hospital, Chinese Academy of Medical Sciences, Beijing 100730, China; 4 华中科技大学同济医学院附属协和医院，武汉 430022 Union Hospital, Tongji Medical College, Huazhong University of Science and Technology, Wuhan 430022, China; 5 海军军医大学第二附属医院（上海长征医院），上海 200003 The Second Affiliated Hospital of Naval Medical University （Shanghai Changzheng Hospital）, Shanghai 200003, China; 6 中国医科大学附属盛京医院，沈阳 110004 Shengjing Hospital of China Medical University, Shenyang 110004, China; 7 上海交通大学医学院附属瑞金医院，上海 200025 Shanghai Institute of Hematology, State Key Laboratory of Medical Genomics, National Research Center for Translational Medicine at Shanghai, Ruijin Hospital Affiliated to Shanghai Jiao Tong University School of Medicine, Shanghai 200025, China; 8 四川大学华西医院，成都 610044 West China Hospital of Sichuan University, Chengdu 610044, China; 9 郑州大学第一附属医院，郑州 450000 The First Affiliated Hospital of Zhengzhou University, Zhengzhou 450000, China; 10 南方医科大学南方医院，广州 510515 ^10^Nanfang Hospital, Southern Medical University, Guangzhou 510515, China; 11 河北大学附属医院，保定 071030 The Affiliated Hospital of Hebei University, Baoding 071030, China; 12 北京大学第三医院临床流行病学研究中心，北京 100083 Peking University Third Hospital, Beijing 100083, China

**Keywords:** 华氏巨球蛋白血症, 医师调研, 横断面研究, 诊疗现状, Waldenström macroglobulinemia, Physician survey, Cross-sectional study, Diagnosis and treatment understanding

## Abstract

**目的:**

深度了解我国临床医师对华氏巨球蛋白血症（WM）疾病的认知，临床诊疗行为和经验，为促进我国WM规范化诊疗，改善WM患者临床结局提供研究证据。

**方法:**

开展面向全国多家三级以及二级医院内血液科、血液肿瘤科以及肿瘤内科医师的调研，自2022年2月至2022年7月招募有WM诊疗经验的临床医师，使用定性序贯定量调研的方法开展研究。

**结果:**

来自于22个省级行政区内33个城市中219家医院的415位临床医师参加了调研。调研结果显示，在诊断方面，虽然医师为疑似WM患者开具的检查检验项目较为统一（实验室检查项目建议率92％～99％、病理检查79％～95％、基因检查96％、影像学检查63％～83％），但在临床实践中（医师认为）仍有22％的患者会被误诊为其他疾病，且非三甲医院的误诊率高于三甲医院（29％对21％，*P*<0.001），WM极易与其他疾病混淆以及医师经验不足无法做出准确判断是医师认为的最主要原因；96％的医师认为WM患者需接受MYD88和CXCR4为主的基因检测，因其有助于疾病确诊以及指导治疗方案的选择。在治疗方面，55％的医师认为缓解症状是主要治疗目标，另外检查指标的改善（54％）以及延长总生存期（51％）也是我国医师关注的治疗目标。在有治疗指征的患者中，医师认为21％左右的患者不会接受治疗，主要是经济因素以及患者对疾病认知不足造成的。在选择治疗药物时，63％的医师会把患者是否可以负担治疗药物作为主要的影响因素，其次是患者合并症（61％）、基因检测结果（55％）、疾病风险等级（54％）等。在治疗方案选择上无论是初诊患者还是复发/难治患者，94％医师认为布鲁顿酪氨酸激酶抑制剂（Bruton tyrosine kinase inhibitors, BTKi）是WM最主要的治疗药物（初治95％，复发75％），BTKi中伊布替尼推荐比例最高（84％）。对于接受治疗的患者中，医师的认为约23％的患者不能完成计划的治疗方案，主要原因与有治疗指征但未接受治疗的原因相同。针对WM的学科发展，66％的医师认为仍需加强临床医师和患者对疾病认知，提高WM的诊断率。

**结论:**

本研究是首项针对WM的全国范围医师调研，系统性地描述了我国医师在WM诊疗中存在的问题，包括疾病误诊率高、基因检测和治疗新药可及性低、患者对治疗依从性差，我国医师认为改善医师和患者对WM的认识是当前亟须解决的问题之一。

## Abstract (en)

**Objective:**

To conduct a nationwide physician survey to better understand clinicians’ disease awareness, treatment patterns, and experience of Waldenström macroglobulinemia （WM） in China.

**Methods:**

This cross-sectional study was conducted from February 2022 to July 2022 by recruiting clinicians with WM treatment experience from hematology, hematology-oncology, and oncology departments throughout China. Quantitative surveys were designed based on the qualitative interviews.

**Results:**

The study included 415 clinicians from 219 hospitals spread across thirty-three cities and twenty-two provinces. As for diagnosis, the laboratory tests prescribed by physicians for suspected WM patients were relatively consistent （92％–99％ recommendation for laboratory, 79％–95％ recommendation for pathology, 96％ recommendation for gene testing, and 63％–83％ recommendation for imaging examination）. However, from a physician's perspective, there was 22％ misdiagnosis occurred in clinical practice. The rate of misdiagnosis was higher in lower-level hospitals than in tertiary grade A hospitals （29％ *vs* 21％, *P*<0.001）. The main reasons for misdiagnosis were that WM was easily confused with other diseases, and physicians lacked the necessary knowledge to make an accurate diagnosis. In terms of gene testing in clinical practice, 96％ of participating physicians believed that WM patients would require gene testing for MYD88 and CXCR4 mutations because the results of gene testing would aid in confirming diagnosis and treatment options. In terms of treatment, 55％ of physicians thought that the most important goal was to achieve remission, while 54％ and 51％ of physicians wanted to improve laboratory and/or examination results and extend overall survival time, respectively. Among patients with treatment indications, physicians estimated that approximately 21％ of them refused to receive treatment, mainly owing to a lack of affordable care and disease awareness. When selecting the most appropriate treatment regimens, physicians would consider patient affordability （63％）, comorbidity （61％）, and risk level （54％）. Regimens containing Bruton tyrosine kinase inhibitor （BTKi） were most widely recommended for both treatment-naïve and relapsed/refractory patients （94％ for all patients, 95％ for treatment-naïve patients, and 75％ for relapsed/refractory patients）, and most physicians recommended Ibrutinib （84％）. For those patients who received treatment, physicians reported that approximately 23％ of patients did not comply with the treatment regimen due to a lack of affordability and disease awareness. Furthermore, 66％ of physicians believe that in the future, increasing disease awareness and improving diagnosis rates is critical.

**Conclusion:**

This study is the first national physician survey of WM conducted in China. It systematically describes the issues that exist in WM diagnosis and treatment in China, such as a high rate of misdiagnosis, limited access to gene testing and new drugs, and poor patient adherence to treatment. Chinese doctors believe that improving doctors’ and patients’ understanding of WM is one of the most urgent issues that must be addressed right now.

华氏巨球蛋白血症（Waldenström macroglobulinemia, WM）是一种罕见的惰性B细胞淋巴瘤，以产生单克隆免疫球蛋白M（IgM）的克隆性淋巴浆细胞浸润骨髓为特征。欧美国家对此病的流行病学、发病机制、诊断、治疗及管理都进行了系统性的研究，数据显示WM在非霍奇金淋巴瘤（NHL）中占比<2％[1]，且欧美人群WM发病率高于亚洲人群[2]–[3]；我国一项单中心回顾性研究结果显示，WM患者在B细胞慢性淋巴增殖性疾病（B-CLPD）中约占13％[4]，较低的发病率导致我国医师对这一疾病的研究甚至诊疗经验都较为有限，且因WM疾病特征广泛，需要与单克隆免疫球蛋白血症、多发性骨髓瘤，以及其他B细胞增殖性疾病鉴别，排他性诊断加剧了医师对WM的诊断上的挑战。随着《淋巴浆细胞淋巴瘤/华氏巨球蛋白血症诊断与治疗中国专家共识》在2016年发布[5]，一定程度上指导了临床医师对这一疾病的管理，但目前我国医师对WM治疗的认知及诊疗现状如何，缺乏相关数据的报道。

因此，本研究目的为通过开展全国范围内的医师调研，帮助深度了解临床医师对WM疾病的认知，诊疗行为和经验，为后续制定更具中国国情的WM指南提供依据，从而促进我国WM规范化诊疗，提高患者治疗疗效。

## 对象与方法

一、研究对象

本研究自2022年2月至2022年7月面向全国三级甲等、三级乙等以及二级医院内血液科、淋巴瘤内科、血液肿瘤科以及肿瘤内科的临床医师发起招募，采取自愿的原则进行调研。入组标准为医师职称在主治医师级别或以上，且有既往诊疗至少1例WM患者的经验。

二、研究方法

本研究使用定性序贯结合定量调研的方法，即首先采用半定式（semi-structured）访谈形式进行定性调研，为定量阶段提供参考变量，调研时长为60 min。随后通过问卷形式开展定量调研，即受访医师根据其诊疗经验填写问卷，调研时长为45 min。调研中涉及的问题及变量主要分为参研医师基本信息、疾病诊断、基因检测应用情况、治疗模式、患者管理、医师获取信息渠道及未来发展趋势六个方面。

本研究在开展前已获得中国医学科学院血液病医院伦理委员会审查批准（批件号：QTJC2022005-EC-1），且每位受访者在参加调研前均已签署知情同意书。调研中收集的信息均已去除受访者身份识别信息，并给予每位受访者独立的研究ID。

三、统计学处理

调研中收集的数据分为分类变量和连续变量。分类变量描述为频数和构成比；连续性变量将用*x*±*s*或*M*（IQR）描述。除总体分析外，研究将对比三甲医院与非三甲医院，以及一年内接诊≤4例WM患者和>4例WM患者的医院之间可能产生的差异。两组间分类变量将采用卡方检验或Fisher精确检验的方法；连续变量将采用独立样本*t*检验或秩和检验进行差异的假设检验。*P*<0.05表示差异具有统计学意义。

## 结果

一、研究对象基本信息

共415位临床医师参加调研，其中15位参与定性调研，400位参与定量调研。调研对象来自于22个省级行政区内33个城市中的219家医院，其中187家医院为三甲医院，其余医院为三级乙等医院或二级医院（以下简称为非三甲医院）（表1）。

**表1:** 定量调研对象分布

省份	医院数量（家）			医院样本量（份）		
	三甲	非三甲	合计	三甲	非三甲	合计
广东	22	1	23	32	13	45
上海	16	7	23	42	1	43
北京	13	9	22	20	15	35
山东	14	1	15	29	2	31
江苏	14	1	15	27	1	28
浙江	14	2	16	24	3	27
福建	9	3	12	13	7	20
湖北	8	4	12	11	8	19
辽宁	8	2	10	14	4	18
重庆	10	0	10	18	0	18
四川	9	0	9	18	0	18
湖南	9	0	9	13	2	15
陕西	8	0	8	14	0	14
吉林	6	1	7	13	0	13
安徽	6	0	6	11	0	11
河南	4	1	5	10	1	11
河北	4	0	4	8	0	8
云南	4	0	4	8	0	8
黑龙江	3	0	3	7	0	7
天津	3	0	3	5	0	5
贵州	2	0	2	4	0	4
江西	1	0	1	2	0	2
合计	187	32	219	343	57	400

参与调研的医师中，86％医师来自血液科，14％来自血液肿瘤科或肿瘤内科。职称方面，28％的医师为主任医师，37％的医师为副主任医师，35％的医师为主治医师。参研医师的中位执业年限为19（IQR 12）年。WM诊疗经验方面，年均诊疗WM患者范围在1～85例，中位数为4（IQR 5）例，诊疗经验中位年限为15（IQR 11）年。

二、诊断现状

研究结果提示，绝大多数医师（97％）会为患者进行危险分级，84％的医师会选择基因检测作为主要的危险分级评价因素，其次为IgM水平（81％）、年龄（79％）、β_2_-微球蛋白水平（79％）以及美国东部肿瘤协作组（ECOG）评分（79％）等。医师认为约75％的WM患者会首诊于血液科或血液肿瘤科，但其他25％患者在其他科室首诊，包括肾内科（7％）、风湿免疫科（6％）、神经内科（5％）以及消化内科（4％）。

首诊时，医师对不同实验室检查项目的建议率在92％～99％（血清蛋白电泳98％、免疫球蛋白97％、免疫固定电泳93％、血常规99％、尿常规92％），病理检查79％～95％（骨髓涂片93％、活检95％、流式细胞术95％、淋巴结活检79％），基因检测96％，不同影像学检查项目在63％～83％（CT检查83％、B超检查79％、PET-CT检查63％）。不同等级以及不同诊疗量的医院中对于辅助检查检验方式的使用比例差异无统计学意义（表2）。

**表2:** 不同等级医院中医师对各类检查或检验的建议情况^a^（％）

检查项目		三甲医院医师	非三甲医院医师	年诊疗量>4例	年诊疗量≤4例
实验室检查	免疫球蛋白（IgM, IgA, IgG）	99	95	99	99
	免疫固定电泳（IFE）	97	96	97	97
	血清蛋白电泳（SPE）	94	89	96	90
	血常规	99	98	99	99
	尿常规	93	89	91	94
病理检查	流式细胞术	96	91	97	94
	骨髓活检	95	96	97	94
	骨髓涂片	94	91	97	91
	淋巴结活检	80	72	76	78
基因检测	基因检测	96	95	98	95
影像学检查	CT	84	79	77	82
	B超	81^b^	67	81	80
	PET-CT	64	56	71	64

注 ^a^ 三甲/非三甲医师为415位，回答年诊疗量的医师为225位（回答>4例的医师为112位，≤4例的医师为113位）；^b^ *P*<0.05

虽然医师为疑似WM患者开具的检查检验项目较为统一，但医师认为仍有22％的WM患者会被误诊为其他疾病。亚组分析中，非三甲医院医师认为的误诊率高于三甲医院医师（29％对21％，*P*<0.001），诊疗量较少（≤4例）医师误诊率与诊疗量较多的医师间差异无统计学意义（22％对21％，*P*＝0.300）；医师认为WM极易与其他疾病混淆以及医师经验不足无法做出准确判断是误诊的最主要原因。

三、基因检测应用情况

研究发现，96％的医师都认为WM患者需接受MYD88和CXCR4为主的基因检测，因其有助于疾病确诊以及指导治疗方案的选择。76％的医师会选择外送基因检测。针对基因检测的方式，60％的医师表示检测的方式为二代测序（NGS），其他医师（40％）表示基因检测方式为PCR。从基因检测的接受程度来说，医师认为约81％的患者接受了基因检测，其中来自三甲医院的患者接受率显著高于非三甲医院的患者（83％对69％，*P*＝0.001）。对于基因检测存在的未被满足需求，82％的医师认为目前基因检测价格过高；76％医师所在医院中的基因检测需要外送进行，导致基因检测送检不便及测序周期长等问题；另外医师认为包括检查结果准确性低（18％）、可能存在假阴性的情况等因素都限制了基因检测的应用。

四、治疗模式

研究发现，55％的参研医师认为症状缓解、检查指标的改善（54％）以及延长总生存（OS）期（51％）是WM患者的主要治疗目标。亚组分析中，更多来自于三甲医院的医师认为延长患者OS期为主要的治疗目标之一（50％对30％，*P*＝0.005），详见图1。

**图1:**
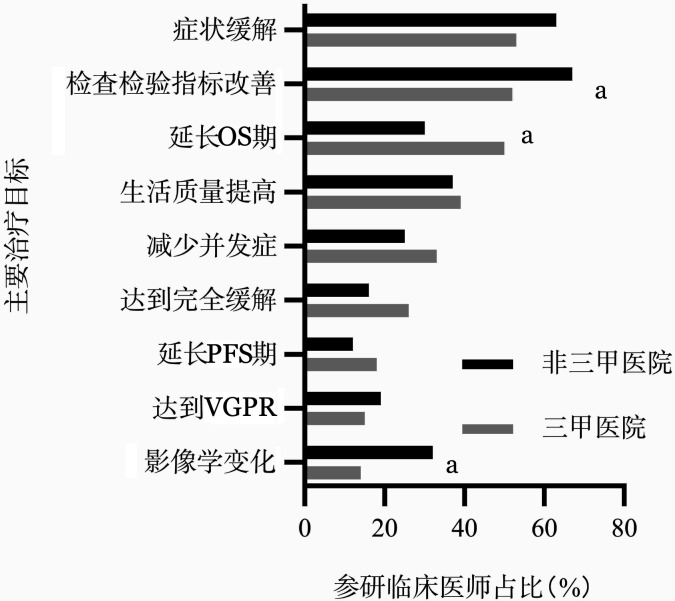
不同等级医院中医师对于华氏巨球蛋白血症的治疗目标 注 OS：总生存；PFS：无进展生存；VGPR：非常好的部分缓解；^a^*P*<0.05

参研医师表示，在有治疗指征的患者中，21％的患者未接受治疗。未接受治疗的原因主要为患者经济因素，以及患者对疾病认知不足。

针对接受治疗的患者，在治疗方案的选择上，63％的医师将患者的经济状况作为最主要的考量因素，其次为合并症（61％）、基因检测结果（55％）、危险度分层（54％）等。进而研究中询问了医师在临床上常为初诊及复发/难治患者推荐的用药方案（表3、4）。研究发现，无论是初诊患者还是复发/难治患者，94％的医师认为布鲁顿酪氨酸激酶抑制剂（BTKi）是WM最主要的治疗药物，针对初诊患者，三甲医院医师为95％，非三甲医院医师为82％（*P*＝0.02）；针对复发/难治患者，三甲医院医师为77％，非三甲医院为58％（*P*＝0.01）；三甲医院对BTKi的建议比例显著高于非三甲医院；在所有BTKi中伊布替尼建议比例最高（84％）。针对复发/难治患者，诊疗量较高的医院建议BTKi +利妥昔单抗（R）方案比例显著高于诊疗量较低的医院（67％对47％，*P*＝0.02）。

**表3:** 医师为初诊华氏巨球蛋白血症患者建议用药方案比例^a^（％）

用药方案	总体（415例）	三甲医院医师	非三甲医院医师	年诊疗量>4例	年诊疗量≤4例
BTK抑制剂	94	95^b^	82	95	91
BTK抑制剂+R	31	31	26	38	28
BR	67	69^b^	54	73	70
硼替佐米+R/BRD	31	32	26	27	37
RCP/RCD	31	33^b^	16	36	30
R	29	29	28	42	28
FR/FCR	21	21	21	14	21
FC	28	27	32	28	28
苯丁酸氮芥	17	16	23	12	14
其他	4	4	<1	3	3

注 BTK：布鲁顿酪氨酸激酶；BR：苯达莫司汀+利妥昔单抗（R）；BRD：硼替佐米+R+地塞米松；RCP：R+环磷酰胺+泼尼松；RCD：R+环磷酰胺+地塞米松；FR：氟达拉滨+R：FCR：氟达拉滨+环磷酰胺+R；FC：氟达拉滨+环磷酰胺；^a^ 题目设置为多选，医师可最多选择三项推荐方案，因此比例总和不为100％，回答了年诊疗量的医师为225位；^b^ *P*<0.05

**表4:** 医师为复发/难治华氏巨球蛋白血症患者推荐用药方案比例^a^（％）

用药方案	总体（415例）	三甲医院医师	非三甲医院医师	年诊疗量>4例	年诊疗量≤4例
BTK抑制剂	75	77^b^	58	79	70
BTK抑制剂+R	54	54	56	67^b^	47
BR	64	63	72	72	64
硼替佐米+R/BRD	31	32	26	29	26
RCP/RCD	23	23	23	29	22
R	14	15	11	15	14
FR/FCR	17	17	19	22^b^	10
FC	15	15	16	14	10
苯丁酸氮芥	9	9	4	5	9
其他	4	3	7	3	2

注 BTK：布鲁顿酪氨酸激酶；BR：苯达莫司汀+利妥昔单抗（R）；BRD：硼替佐米+R+地塞米松；RCP：R+环磷酰胺+泼尼松；RCD：R+环磷酰胺+地塞米松；FR：氟达拉滨+R； FCR：氟达拉滨+环磷酰胺+R； FC：氟达拉滨+环磷酰胺；^a^ 题目设置为多选，医师可最多选择三项推荐方案，因此比例总和不为100％；回答了年诊疗量的医师为225位；^b^ *P*<0.05

在用药依从性方面，医师认为约23％的患者会中断治疗，主要原因是经济因素以及患者认为疾病已经好转，无须继续治疗；BTKi作为主要的治疗药物，医师认为约25％的初诊患者和28％的复发/难治患者会中断BTKi的治疗。相较于三甲医院，非三甲医院医师认为更多患者会停药（初诊：33％对24％，*P*＝0.001；复发/难治：37％对26％，*P*＝0.001）；相较于年诊疗量大的医院，年诊疗量较小医院的医师认为的患者停药比例更高（27％对21％，*P*＝0.010）。

五、患者随访与管理

患者随访方面，无论是新诊断患者还是复发/难治患者，医师推荐正在接受治疗的患者每隔0.5～2个月随访1次，大多数患者（>80％）都会按时随访；未治疗或暂无治疗指征的患者随访间隔较长（2～3个月），实际随访的患者也低于接受治疗患者。绝大多数医师（90％）表示在临床中会做患者管理，主要方式为电话回访（55％）及患者微信群（32％）。值得注意的是， 63％医师表示其所在科室内有针对WM疾病的患者管理项目，且三甲医院比例高于非三甲医院（73％对57％，*P*＝0.02），年诊疗量≥4例的医院比例也较高（84％对63％，*P*＝0.001）。医师所在科室没有针对WM疾病开展患者管理的主要原因包括WM患者数量较少，且并非所有患者都需要治疗或随访（72％），临床工作较忙，没有足够时间做患者管理（54％），缺少高效患者管理项目（49％）和第三方患者管理机构（24％）。

六、医师获取信息渠道

总体来说，几乎所有医师（99％）都会通过指南或专家共识获取针对WM疾病诊疗的相关信息，其中NCCN指南［重要分数（8.62±1.04）分，总分10分］及《淋巴浆细胞淋巴瘤/华氏巨球蛋白血症诊断与治疗中国专家共识（2016年版）》[5]［重要分数（8.31±1.21）分，总分10分］是医师普遍认为最具参考性的临床指南。

七、WM临床实践中未满足的需求以及亟待解决的问题

研究结果显示，医师认为WM疾病领域中仍存在显著的未满足需求及未来亟待解决的问题分别为：医师及患者对WM疾病认知不足（66％），WM疾病诊断率较低，容易发生误诊或漏诊，延误治疗时机（60％），患者管理意识不足（46％），基因检测可及性低（40％），WM治疗方案的疗效（39％）、安全性（24％）和便利性（13％）尚需进一步改善。

## 讨论

WM作为一种较为罕见的NHL，具有发病率低、异质性强、预后差异较大等特征，目前我国针对WM的大样本多中心临床研究或真实世界研究非常有限，导致该疾病管理及规范化诊疗水平落后于其他更为常见的淋巴瘤或血液疾病。本研究收集了我国内地22个省级行政区415位临床医师的调研结果，较全面地体现了WM疾病在我国的疾病认知和诊疗行为。从行政区域来看，参加调研的22个省级行政区分布于我国东北、华北、华中、华东、华南和西南地区，具有较好的区域代表性。

本次研究结果提示，WM的误诊率较高（22％），这种现象在非三甲医院尤为突出（29％）。66％的医师反馈对疾病认识不够，除了低发病率，医师认为WM容易与其他疾病混淆，或因患者检查指标异常不明显，常无法做出准确判断。2022年发布的中国指南[6]对WM鉴别诊断流程做了详细的阐述，在本研究中，医师对于标准鉴别诊断项目的使用与指南推荐较为符合，但仍有医师在确诊流程缺少部分检查检验（例如：并非所有医师都为患者进行病理检查）。因此，WM作为较为罕见的疾病，未来仍需提高医师对于疾病鉴别诊断的认知，尤其需要加强基层医师培训，进而使WM疾病诊治流程更加规范化和标准化。另一方面，西方国家对WM的规范诊疗一定程度上得益于成熟的多学科诊疗模式（MDT），通过MDT合作，可以实现血液科和病理科、影像科等专科之间的充分交流，最大程度减少误诊误治，达到规范和个体化诊疗目的，改善患者结局[7]；加强院内MDT建设也是规范WM诊治的方向。

基因检测在WM的诊疗中起到重要作用，数据显示，西方国家在常规临床实践中广泛实施MYD88突变分析[8]，本次调研发现，目前我国基因检测的可及性和规范性仍存在未被满足的需求，并非所有患者（19％）都会接受基因检测，其中来自非三甲医院的医师认为患者对基因检测的接受率更低（83％对69％，*P*＝0.001），可能与检测价格高、对基因检测认知低、不接受院外检测、测序时间长等有关；我国医师在临床实践中应重视基因检测的诊断价值，预后价值和对治疗方案选择的指导价值，积极进行患者教育，进而提高检测率。

对于WM的治疗目标，中国及国外指南均强调症状缓解是WM的首要目标[6],[9]，而不是缓解深度；本次调研中55％的参研医师认为缓解症状、检查指标的改善（54％）以及延长OS期（51％）是WM患者的主要治疗目标；在一项针对荷兰医师对于WM诊疗认知的调研中，受访者将“减少疾病相关症状”列为最重要的治疗目标（44％）[8]，可以看出对于WM的治疗目标上，我国医师和国际的认识相对统一。

在中国指南中，对于有治疗指征的患者，最高推荐等级治疗方案为含有BTKi的方案[6]。本次研究结果显示，虽然BTKi是医师最主要的治疗药物（94％），但（免疫）化疗在临床实践中仍然是医师推荐的主流方案之一。一项利用美国退伍军人医疗数据库（VHA）大型真实世界研究发现，自BTKi在2013年上市以后，（免疫）化疗的使用率迅速下降，BTKi成为最主要的治疗药物，>70岁WM患者的死亡/疾病风险下降41％[10]；随着以伊布替尼为首的BTKi自2017年开始陆续在中国上市并纳入国家医保报销范围，未来应进一步推广指南在临床实践中的应用价值，推动WM治疗逐渐转变为以疗效和安全性特征更好的靶向药物为代表的无化疗时代，进而帮助规范化治疗体系的建设。

另外在用药依从性方面，我国患者的依从性相对较差[11]，医师认为约23％的患者会中断治疗，BTKi作为临床推荐的首选方案之一，医师认为约25％～28％的患者会中断治疗，其中非三甲医院医师认为患者停药的比例会更高（初诊：33％对24％，*P*＝0.001；复发/难治：37％对26％，*P*＝0.001）。已有研究证明伊布替尼中断治疗会给患者带来IgM及血黏度升高等症状，进而缩短患者生存期[12]，尤其是起始BTKi治疗6个月内停药，患者预后极差[12]，此外，一项中位随访期为59个月的研究中，伊布替尼单药治疗复发/难治WM的总缓解率为90.5％，5年OS率也达到了87％[13]，提示规范的BTKi用药可有效帮助医师和患者达成缓解症状、改善检查检验指标以及延长OS期的治疗目标。因此，在临床实践中，临床医师应强调BTKi长期使用的临床疗效及使用习惯，加强患者对WM疾病的认知。

研究发现，提高WM患者对疾病的认识也是需要解决的问题之一。根据本次调研结果，医师认为高达21％有治疗指征的WM患者未接受治疗，即使接受治疗的患者也有约23％的患者会中断治疗，患者对疾病认知不足是主要的原因之一。因此，未来应加以关注并鼓励更多患者进行治疗，以改善WM患者的预后及延长生存期。另外，在患者管理方面，尽管大多数医师表示会通过回访电话或微信群内开展患者管理，但仅有63％医师表示其所在科室内有针对WM疾病的患者管理项目，非三甲医院的比例更低，因此未来可加强针对WM的患者管理项目建设，提高患者依从性，以改善长期预后。

本研究仍然存在一定局限性，由于调研能力有限，研究未能覆盖我国所有省级行政区及城市，以及招募对象仅来自中国部分三甲医院及非三甲医院的血液科、血液肿瘤科和肿瘤内科，导致研究结果仍然有一定程度上的选择偏倚。另外，对于疾病的诊断及治疗模式均来自于医师的主观报告，未来仍然需要大样本多中心研究来证实真实世界中WM的诊疗现状。然而，本研究是我国首个针对WM领域内临床医师的调查研究，填补了我国临床医师对于疾病认知和诊疗现状的研究空白，基于本研究结果有助于提高医师对于WM管理的认识，推动中国WM指南的制定，进而改善我国医师对WM的规范化诊疗，提高患者治疗疗效，改善患者预后。
